# Susceptibility to antibiotics in isolates of *Lactobacillus plantarum* RAPD‐type Lp299v, harvested from antibiotic treated, critically ill patients after administration of probiotics

**DOI:** 10.1002/mbo3.642

**Published:** 2018-05-24

**Authors:** Bengt Klarin, Anders Larsson, Göran Molin, Bengt Jeppsson

**Affiliations:** ^1^ Department of Anaesthesiology and Intensive Care Lund University and Skåne University Hospital Lund Sweden; ^2^ Department of Surgical Sciences Section of Anaesthesiology and Intensive Care Uppsala University Hospital Uppsala Sweden; ^3^ Applied Nutrition and Food Chemistry Lund University Lund Sweden; ^4^ Department of Surgery Lund University and Skåne University Hospital Malmö Sweden

**Keywords:** antibiotic pressure, antibiotic susceptibility, critically ill patients, E‐test, *Lactobacillus plantarum* 299v, probiotics

## Abstract

Recultured *Lactobacillus plantarum* 299v‐like strains were tested regarding antibiotic susceptibility, and no decrease was detected. Antibiotics are frequently used to treat patients in intensive care units (ICUs) and are associated with a significant risk of selection of resistant bacterial strains. In particular, it is possible that genetic transfer of antibiotic resistance to the resident gastrointestinal flora, as well as to administered probiotics, may be increased in the ICU setting. The aim of the present investigation was to detect possible changes in antimicrobial susceptibility in reisolates of the probiotic strain *Lactobacillus plantarum* 299v (Lp299v) given to antibiotic treated, critically ill patients. Lp299v‐like strains were identified in cultures of biopsies and fecal samples from 32 patients given the probiotic strain enterally in two previous ICU studies. The patients received a variety of antibiotics. Isolates with the same genomic RAPD profile (RAPD‐type) as Lp299v were obtained to enable monitoring of antibiotic susceptibility by E‐tests. Forty‐two isolates, collected throughout the course of illness, were tested against 22 different antibiotics. No obvious decrease in susceptibility was found for 21 of the tested antibiotics. There was a tendency toward decreased susceptibility to ampicillin. The stable antibiotic susceptibility profiles of the Lp299v‐like isolates studied here suggests this probiotic is less likely to acquire resistance when administered to critically ill patients treated with broad‐spectrum antibiotics.

## 1 INTRODUCTION

Probiotics are widely used in society for health promotion, and they are often employed in medical settings, primarily to prevent the side effects of antibiotics. A number of studies and reviews have suggested that probiotics have beneficial effects on critically ill patients (Barraud, Bollaert, & Gibot, 2013; Bo et al., 2014; Elaine et al., 2012; Goldenberg et al., 2013; Manzanares, Margot Lemieux, Langlois, & Wischmeyer, 2016; Shimizu et al., 2013), whereas other investigations have shown no advantages of probiotics compared to controls (Gu, Wei, & Yin, 2012; Wang et al., 2013). However, little is known about a number of critical issues in this context, such as the possible impact of probiotics on the normal microbiological flora (Imperial & Ibana, 2016), and the effects of administered drugs on the antibiotic susceptibility of probiotics. Some experimental animal data support the suggestion that antibiotic resistance genes are transferred to probiotic strains (Mater, Langella, Gérard Corthier, & Flores, 2008), although this aspect has not been studied in the hospital environment.

The highest antibiotic pressure is in the intensive care units (ICUs), where patients with few exceptions are given antibiotics, mainly broad‐spectrum agents, as an important part of their treatment. Therefore, in ICUs there is significant ongoing selection of bacteria with resistance to antibiotics (Karam, Chastre, Wilcox, & Jean‐Louis Vincent, 2016; Zilahi, Artigas, & Martin‐Loeches, 2016). The colon can be regarded as a fermenter that has a high bacterial content, and it is highly likely that there is exchange of different molecules including genetic material. Probiotics are used in many ICUs mainly in order to reduce the prevalence of antibiotic‐associated diarrhea. Nonetheless, we hypothesized that transfer of antibiotic resistance to probiotics could occur in this context.

*Lactobacillus plantarum* is considered to be a genomically stabile species. Furthermore, regular tests of the in vitro antimicrobial susceptibility of Lp299v to a number of agents have failed to reveal any changes over the years. Guidelines on interpretive breakpoints for the minimum inhibitory concentration (MIC) values of a number of antibiotics against strains of the species *Lactobacillus plantarum* have been published by the European Food Safety Agency (EFSA) (EFSA 2005) and were updated for some of these agents in 2008 (EFSA 2008) and 2012 (EFSA 2012).

*Lactobacillus plantarum* 299v (Lp299v; DSM9843) is the probiotic component of a number of products that have been commercially available for more than three decades in Sweden and for several years in other countries. Moreover, Lp299v in the form of a fruit drink (ProViva^®^) has been used as a prophylactic remedy in many Swedish hospitals, including ICUs. To date, several ICU studies using the strain Lp299v (Klarin, Johansson, Molin, Larsson, & Jeppsson, 2005; Klarin, Wullt, et al., 2008; McNaught, Woodcock, Anderson, & MacFie, 2005), and the genomically closely related *L. plantarum* 299 (= DSM 6595) [Klarin, Molin, Jeppsson, & Larsson, 2008; Rayes, Seehofer, et al., 2002; Rayes, Hansen, et al., 2002) have not shown any important side effects of these probiotics.

Two earlier ICU‐based studies of critically ill patients receiving broad‐spectrum antibiotics addressed the following issues: (1) whether administration of Lp299v resulted in adherence of the probiotic to the gut mucosa (Study 1, Klarin et al., 2005) and (2) whether coadministration of Lp299v with antibiotics reduced colonization of *Clostridium difficile* (Study 2, Klarin, Wullt, et al., 2008). In the present investigation, 42 isolates of RAPD‐type Lp299v cultured from rectal mucosal biopsies (Study 1) or fecal specimens (Study 2) were investigated to determine whether antibiotic susceptibility profiles were altered after gastrointestinal transit in patients on broad‐spectrum antibiotics. The results showed no significant changes in the antibiotic susceptibility profiles of Lp299v‐like isolates screened against a panel of 22 broad‐spectrum antibiotics. This finding together with the low incidence of side effects observed in Studies 1 and 2, indicates that the probiotic Lp299v should be a viable candidate for use in critically ill patients receiving antibiotics.

## 2 MATERIALS AND METHODS

### 2.1 Samples

**Figure 1:**
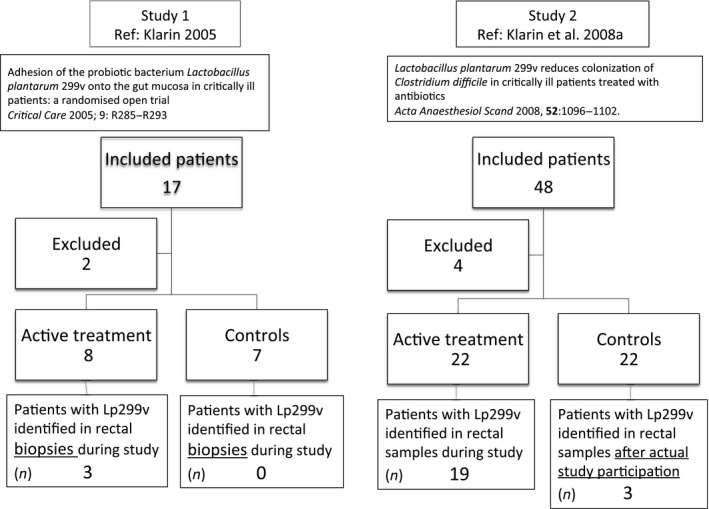
Flow diagram showing the number of patients that participated in Studies 1 and 2

In two separate prospective randomized controlled investigations (Studies 1 and 2) the probiotic strain Lp299v was given enterally to critically ill adult patients anticipated to require intensive care for ≥3 days. Eight patients in Study 1 and 22 patients in Study 2 were given Lp299v. The control groups (seven and 22 patients, respectively) received similar standard treatments but no probiotics (Figure 1). Biopsies were taken in Study 1, and fecal samples were collected in Study 2. Six samples (three from each study) collected before start of the intervention began were positive for Lp299v. All other positive samples shown in the figures came from patients randomized to the Lp299v group. In Study 1, a total of 47 biopsies were taken (27 in the Lp299v group and 20 in the control group), with a median of three samples per patient. In Study 2, 167 fecal samples were collected (74 and 93 in Lp299v and control patients, respectively), also with a median of three samples per patient. Lp299v‐like isolates were obtained from biopsies of the rectal mucosa (Study 1), or from fecal samples (Study 2). In both studies samples were collected at inclusion and thereafter twice a week until discharge from the ICU. The patients were treated with different types of antibiotics, initially empirically and thereafter in accordance with clinical findings and the results of microbiological cultures. All patients had received one or more doses of antibiotics before any study product was given and before the first biopsy or fecal sample.

### 2.2 Analyses

Lactobacilli were cultured from rectal mucosal biopsies or fecal specimens at the time of Studies 1 and 2 using Rogosa agar (Oxoid, Basingstoke, Hampshire, England) incubated anaerobically at 37°C for 3 days. Colonies suspected to be Lp299v (large, creamy white‐yellowish, and somewhat irregular in shape) were isolated and further identified by randomly amplified polymorphic DNA typing (RAPD) (Johansson, Quednau, Molin, & Ahrné, 1995). All isolates were stored at −80°C pending analysis. After reconditioning of the frozen strains, *Brucella* broth suspensions of the respective strain, were inoculated on *Brucella* agar plates (Oxoid). E‐test strips of 22 antibiotics (detailed below in section 3.2) were applied on the inoculated agar plates, and incubated anaerobically at 35°C for 72 hr. All analyses were done in duplicate.

To enable comparisons of MIC values of the harvested isolates and the original strain, the isolates were divided into four groups according to their exposure to antibiotics and administration of Lp299v: (1) isolates cultured from samples collected prior to probiotic intervention in each study; (2) isolates obtained from rectal mucosa biopsies; (3) isolates acquired from fecal samples; (4) isolates obtained from fecal samples from patients in Study 2 given Lp299v (as the fruitdrink ProViva^®^) after conclusion of their participation in the study. The subjects in group 4 were given the control product (without probiotics) during the study period, but then as non‐ICU patients at the same Department of Infectious Diseases received the same treatment as other patients in that ward.

### 2.3 Tested antibiotics

A wide panel of antibiotics (E‐tests; AB Biodisk, Solna, Sweden) was tested against the original strains and the Lp299v‐like isolates. The following antibiotics were given to one or more of the study participants were ampicillin, cefotaxime, ceftazidime, cefuroxime, clindamycin, erythromycin, gentamicin, imipenem, levofloxacin, meropenem, metronidazole, netilmicin, piperacillin, tobramicyn, trimethoprim (to patients given in combination with sulfametoxazole), and vancomycin. To fulfill regulatory requirements for the company holding the patent on Lp299v (Probi AB), some antibiotics seldom used in Sweden were also tested namely; cefepime, chloramphenicol, kanamycin, linezolid, quinupristin/dalfopristin, and streptomycin.

## 3 RESULTS

### 3.1 Studied samples

Forty‐two cultured Lp299v‐like isolates were analyzed together with the original strain (Lp299v), and with the genomically closely related strain *Lactobacillus plantarum* 299.

The strain *L. plantarum* 299 (DSM6595) has been used in studies of ICU patients conducted by our group (Klarin, Molin, et al., 2008) and by other researcher (Oláh, Belágyi, Issekutz, Gamal, & Bengmark, 2002; Rayes, Seehofer, et al., 2002; Rayes, Hansen, et al., 2002), and therefore it was also of interest to evaluate the antibiotic susceptibility of this probiotic. Six isolates were from samples taken at study inclusion (prior to probiotic intervention), and 24 were from samples collected during the study periods. From three patients in the control group (not given Lp299v) in Study 2, 16 samples (12 of them positive for Lp 299v) were obtained after conclusion of the actual study period. These patients required an extended care period with further antibiotic therapy after their stay in ICU, and as ordinary ward patients they received routine care that included the fruit drink ProViva^®^.

### 3.2 MIC determinations

**Figure 2:**
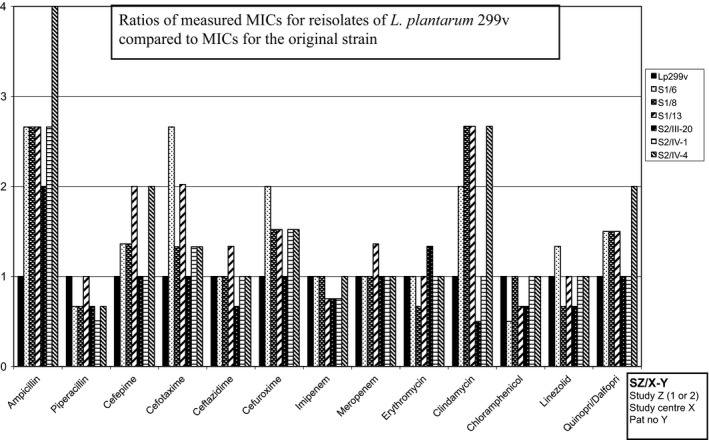
Reisolates of *L. plantarum* 299v found in inclusion samples

**Figure 3:**
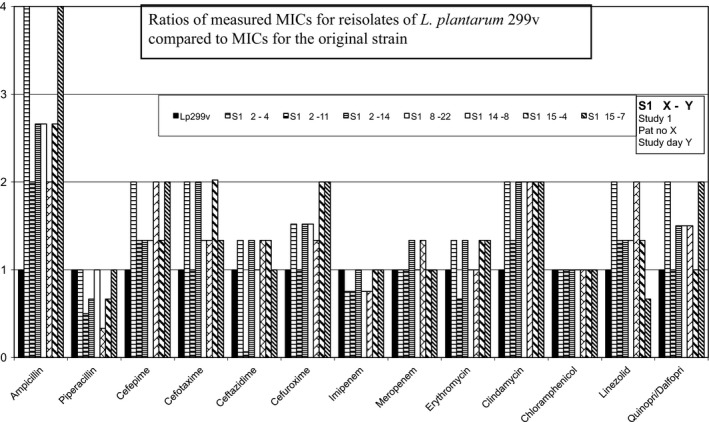
Reisolates of *L. plantarum 299v* collected in Study 1

**Figure 4:**
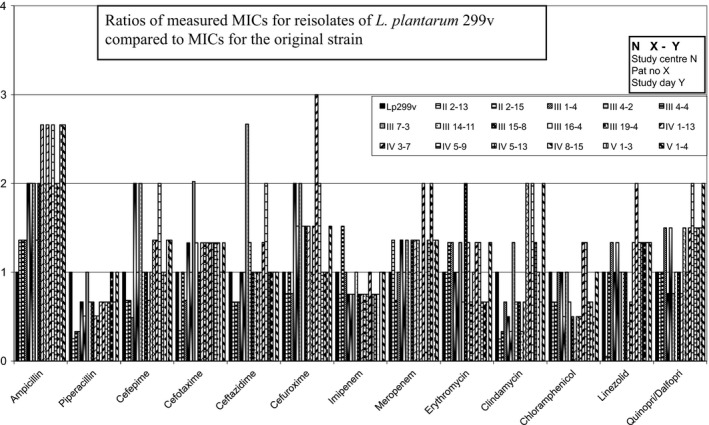
Reisolates of *L. plantarum 299v* collected in Study 2

**Figure 5:**
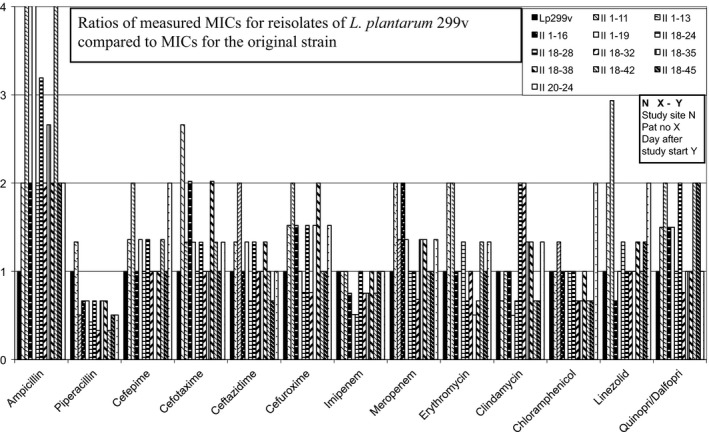
Reisolates of *L. plantarum 299v* collected from control patients after concluding participation in Study 2

The MIC values determined for Lp299v and *L. plantarum* 299 were within a twofold (1 + 1) dilution (Table 1) and thus did not differ measurably. Both Lp299v and *L. plantarum* 299 are intrinsically resistant to aminoglycosides, vancomycin, and metronidazole, and in this study they also had high MIC values for levofloxacin. Ratios of MICs for the remaining 13 antibiotics to MICs of the Lp299v original strain are shown in Figures 2, 3, 4, 5. We found no significant changes in susceptibility to most of the tested drugs. Differences in MIC levels were within a twofold dilution step (1 + 1) except for ampicillin, several isolates of which showed an MIC increase in two 1 + 1 steps (1 + 3). There was no correlation with any increase in MIC for other antibiotics in the samples that had an increased MIC for ampicillin. MIC breakpoints for some of the tested antibiotics have been determined by EFSA (EFSA 2005) (Table 1), but not for antibiotics that are frequently used in ICUs. For the MIC breakpoints that are available, none of the observed deviations from the original strain led to changes from susceptibly to resistance.

**Table 1:** MIC values (mg/L) determined by E‐tests for *L. plantarum* 299 and *L. plantarum* 299v, and MIC breakpoints for the species *L. plantarum* presented by EFSA (EFSA 2005)

Antibiotic	*L. plantarum* 299	*L. plantarum* 299v	EFSA, MIC breakpoints species *L. plantarum*
Ampicillin	0.094	0.094	4
Cefepime	0.047	0.047	Not specified/tested
Cefotaxime	0.094	0.094	Not specified/tested
Ceftazidime	0.5	0.75	Not specified/tested
Cefuroxime	0.25	0.5	Not specified/tested
Chloramphenicol	2	2	8
Clindamycin	2	3	4
Erythromycin	0.75	1	4
Gentamicin	32	32	64
Imipenem	0.064	0.064	Not specified/tested
Kanamycin	>256	>256	64
Levofloxacin	32	32	Not specified/tested
Linezolid	1	0.75	4
Meropenem	0.064	0.064	Not specified/tested
Metronidazole	>256	>256	Not specified/tested
Neomycin	Not tested	Not tested	32
Netilmicin	48	32	Not specified/tested
Piperacillin	0.5	0.75	Not specified/tested
Quinupri/Dalfopri	0.5	0.5	4
Streptomycin	>256	>256	64
Tetracycline	Not tested	Not tested	32
Tobramycin	>256	>256	Not specified/tested
Trimethoprim	0.125	0.125	8
Vancomycin	>256	>256	`Not required`

MIC, minimum inhibitory concentration.

The tested isolates were exposed to different antibiotics and combinations of antibiotics and to the environment in the GI tract for varying periods of time. In almost all cases the drugs were given intravenously, and cephalosporins and carbapenems were used most frequently. In patients with several subsequent isolates, we found no gradual change in susceptibility. Four of the six patients with isolates from the sample taken at study inclusion had been treated with antibiotics for 1–20 days before start of the investigation. Our results demonstrate that the antibiotic susceptibility of the probiotic bacterium *Lactobacillus plantarum* 299v remains stable also after passage through the GI tract in patients treated with broad‐spectrum antibiotics.

## 4 DISCUSSION

The present evaluation was performed on frozen samples that had been collected in two previous studies and stored at −80°C (Studies 1 and 2) and our aim was to detect changes in antibiotic susceptibility of a probiotic *Lactobacillus* strain given to intensive care patients. These patients were treated with broad‐spectrum antibiotics administered parenterally, and hence major changes in the microbiota could be anticipated, in particular selection of antibiotic‐resistant bacterial species or clones. Conceivably, this situation would favor dissemination of antibiotic resistance that would include the probiotic strain in focus in our study. However, in two cohorts of patients, we were unable to detect any changes in susceptibility of the probiotic strain to a number of antibiotics, most of which had been used clinically at our ICU. Furthermore, we found no changes from susceptibility to resistance for any of the tested antibiotics. It was impossible for us to conclude that transitions cannot occur, because MIC breakpoints are lacking for many of the antibiotics widely used to treat critically ill patients. Notwithstanding, the MIC values for Lp299v for the antibiotics considered, (e.g., cephalosporins and carbapenems) were all low, indicating that development of resistance is less likely.

In six of the 42 samples that we assessed (representing five of 32 individuals) the ampicillin MIC values showed an increase in two 1 + 1 dilution steps (1 + 3), possibly approaching what can be considered decreased susceptibility. Consecutive samples from some of those patients showed no trend towards greater reduction in susceptibility, although the values did shift over time (but were nonetheless higher than values for the original strain). If this was indeed a true decrease in susceptibility, there probably would have been an increase over time or a stable increase in the MIC value. None of the patients of interest were treated with ampicillin. The major part of the samples with an increase in the MIC of ampicillin were collected after several days of treatment in the ICU.

Other species and strains used as probiotics show intrinsic resistance to several antibiotics (Wong, Ngu, Dan, Ooi, & Lim, 2015), although with different patterns compared with *L. plantarum* 299 and Lp299v. However, the changes in antibiotic susceptibility of bacterial strains used as probiotics have been studied and have in some cases been found to be due to increased activity in cellular efflux pump mechanisms (Thumu & Halami, 2012). This may explain the observed change in the MIC of ampicillin.

Lp299v and *L. plantarum* 299 have been used in several clinical studies without any reports of infections with pathogens showing extended antimicrobial resistance that might have originated from these two strains. Therefore, we focused the present postexposure survey focused on these probiotics.

The GI tract is estimated to harbor 600 or more bacterial species, some of which exhibit intrinsic or acquired resistance to various antibiotics. Inevitably, such species or strains may be positively selected to varying degrees during antibiotic treatment, as exemplified by the occurrence of antibiotic‐associated diarrhea, which is often caused by the opportunistic pathogen *Clostridium difficile* (Kevin, Brown, Khanafer, Nick Daneman, & Fismana, 2013; Napolitano & Edmiston, 2017; Vardakas, Trigkidis, Boukouvala, & Falagas, 2016). An investigation of pharyngeal streptococci in healthy volunteers demonstrated that even short periods of treatment with macrolides (3 days for azitromycin) were sufficient to increase the proportion of macrolide‐resistant strains from 26% to 86%, and such ecological changes persisted for at least 6 months (Malhotra‐Kumar, Lammens, Coenen, Van Herck, & Goossens, 2007). Also a study of patients admitted for thoracic surgical procedures and given cefazolin for various lengths of time showed a significant increase in the prevalence of resistant *Escherichia coli* at discharge compared to admission (Jonkers, Swennen, London, Driessen, & Stobberingh, 2002). The figures reported in the cited study may not be representative for all species and locations, but they do reveal dramatic changes in selection of resistant strains within a few days of antibiotic treatment. Many of the retrieved isolates in the present investigation came from patients with prolonged antibiotic load, and the probability of either an induced resistance or selection of resistant bacteria would be considered high in such subjects. Gram‐negative bacteria may play the most important role in infections originating from the GI tract, although gram‐positive pathogens are also a clinical issue. Increasing occurrence of multidrug‐resistant cocci is becoming a serious problem in many clinics and above all in critically ill patients (Lee, Lee, Park, Jeong, & Lee, 2015; Munita, Bayer, & Arias, 2015). Selective decontamination has been applied for many years and has been considered not to carry any risk of development of resistant bacteria, although modern techniques have disproved that assumption (Buelow, Bello González, & Verslius, 2014; Buelow et al., 2017). In contrast the use of probiotics has appeared as a complement in the prevention of emergence of antibiotic resistance (Ouwehand, Forssten, Hibberd, Lyra, & Stahl, 2016).

Treatment with antibiotics changes the intestinal microbiota in an unfavorable way, but ongoing research concerning this topic and use of probiotics as a complement in restoring a microbial balance has provided promising results. It is necessary to find suitable commensals of human origin for use in approaches developed to reestablish balance and colonization resistance (Blaser, 2016; Pamer, 2016) is of vital importance. The results of our investigation identify Lp299v, alone or more likely in combination with other strains as a possible candidate for use in this context. Lp299v was originally detected in human feces.

Clearly, it is necessary to consider the question of whether the analyzed strains actually constituted bacteria that had been exposed long enough to the conditions that prevail in the endogenous fermenter—the colon. We have previously demonstrated that Lp299v becomes established on the rectal mucosa to the same extent in antibiotic treated, critically ill patients as in healthy volunteers (Klarin et al., 2005). Introduced exogenous bacteria and fungi (probiotics or other microorganisms) are chiefly visitors to the GI tract, whereas the commensal microbiome is by definition prone to remain. Administered Lp299v bacteria adhere to the mucosa and stay there for shorter or longer periods of time. The mechanism for this adhesion occurs via a mannose entity on the epithelium cell site (Adlerberth et al., 1996) and hence it is plausible that Lp299v first adheres to the mucosa, after which the bonding at that location is disrupted and the bacteria are transported with the luminal content and then adhere at another binding site a bit further down the gut. This process is repeated and might be considered analogous to a chromatography column. In addition, since the Lp299v thrive in the gut, new generations of this strain—with the same genetic profile—should be released. Inasmuch as Lp299v has been found to remain adhered to the rectal mucosa long after cessation of oral administration (Johansson et al., 1993), it is reasonable to assume that the mannose‐to‐bacterium adherence bond is fairly strong. This implies that the exposure time necessary for exchange of genetic material in the GI tract should be sufficient, and also that the feces samples taken can actually can represent exposed bacteria and not merely bacteria that have rapidly passed through the gut.

In our study, the MIC values were not identical for the exposed bacteria and the native strain, which strengthens our argument that the cultured strains represent bacteria that had been properly exposed to the fermentative environment of the GI tract. It is distinctly possible that the retrieved Lp299v bacteria had adhered to the mucosal epithelium several times in different locations during their journey to the rectum or that they were indeed new generations of this strain.

We found no changes in antibiotic susceptibility, and thus we conclude that the Lp299v genome is stable. Therefore it can also be assumed that there is little risk that Lp299v genetic material will spread to other species.

Conditions in the human (and animal) GI tract, and especially in the colon, are suitable for genetic exchange between species (Salyers, Gupta, & Wang, 2004), with some species being more prone than others to act as donors and/or recipients. Furthermore, animal studies have provided overwhelming evidence for in vivo bacterial transfer of resistance genes (Moubareck, Bourgeois, Courvalin, & Doucet‐Populaire, 2003; Tannock, Bateup, & Jenkinson, 1993). It also seems likely that bacteria transiently colonizing the intestine (e.g., probiotics) can take part in the exchange of resistance genes. Accordingly, as part of the safety profile of probiotics in preparations marketed as health products and for prophylactic use in hospitals, it should be confirmed that the bacteria strains in these products are not prone to development of antibiotic resistance, and this should be demonstrated by available in vitro and in vivo methodology.

In conclusion, we found no evidence that *Lactobacillus plantarum* 299v is prone to acquiring genetic material coding for antimicrobial resistance in antibiotic treated, critically ill patients. In the context of susceptibility, *L. plantarum* 299v is also stable against antimicrobial agents in clinical settings with high antimicrobial pressure.

## CONFLICT OF INTEREST

GM and BJ are stock holders in Probi AB.
